# Topical Application of Fibroblast Growth Factor 10-PLGA Microsphere Accelerates Wound Healing via Inhibition of ER Stress

**DOI:** 10.1155/2020/8586314

**Published:** 2020-12-07

**Authors:** Ke Xu, Bo Chai, Kailun Zhang, Jun Xiong, Yiru Zhu, Jingyu Xu, Ningchen An, Weidong Xia, Hao Ji, Yanqing Wu, Hao Li, Jian Xiao, Zhiguo Feng, Hongyu Zhang

**Affiliations:** ^1^School of Pharmaceutical Sciences, Wenzhou Wound Repair and Regeneration Key Laboratory, Cixi Biomedical Research Institute, Wenzhou Medical University, Zhejiang, China; ^2^The Institute of Life Sciences, Engineering Laboratory of Zhejiang Province for Pharmaceutical Development of Growth Factors, Biomedical Collaborative Innovation Center of Wenzhou, Wenzhou University, Zhejiang, China; ^3^Department of Burn, First Affiliated Hospital of Wenzhou Medical University, Zhejiang, China; ^4^Department of Orthopedics Surgery, Lishui People's Hospital, The Sixth Affiliated Hospital of Wenzhou Medical University, Zhejiang, China

## Abstract

There is a high incidence of acute and chronic skin defects caused by various reasons in clinically practice. The repair and functional reconstruction of skin defects have become a major clinical problem, which needs to be solved urgently. Previous studies have shown that fibroblast growth factor 10 (FGF10) plays a functional role in promoting the proliferation, migration, and differentiation of epithelial cells. However, little is known about the effect of FGF10 on the recovery process after skin damage. In this study, we found that the expression of endogenous FGF10 was increased during wound healing. We prepared FGF10-loaded poly(lactic-co-glycolic acid) (FGF10-PLGA) microspheres, and it could sustain release of FGF10 both in vitro and in vivo, accelerating wound healing. Further analysis revealed that compared with FGF10 alone, FGF10-PLGA microspheres significantly improved granulation formation, collagen synthesis, cell proliferation, and blood vessel density. In the meantime, we found that FGF10-PLGA microspheres inhibited the expression of endoplasmic reticulum (ER) stress markers. Notably, activating ER stress with tunicamycin (TM) reduced therapeutic effects of FGF10-PLGA microspheres in wound healing, whereas inhibition of ER stress with 4-phenyl butyric acid (4-PBA) improved the function of FGF10-PLGA microspheres. Taken together, this study indicates that FGF10-PLGA microspheres accelerate wound healing presumably through modulating ER stress.

## 1. Introduction

Wound healing is a complex biological process involving the activation of multiple factors [1–3]. Failure in any link can cause a slow or failed wound healing. The skin wound repair process involves inflammatory response, repair process, extracellular matrix reconstruction, growth factor action, and other links [4, 5]. While less effective repair makes it difficult for the wound to heal, excessive repair usually leads to hypertrophic scars [6]. Recent studies have shown that growth factors, such as those activating endothelial cells to initiate angiogenesis, play a pivotal role in wound healing. FGF10, also known as keratinocyte growth factor 2 (KGF2), has a variety of biological functions and is involved in multiple processes, including angiogenesis, embryo development, and hair growth [7–9]. However, the role of FGF10 in wound healing remains unclear.

It has been reported that FGF10 accelerated wound healing in venous ulcers of clinical trials. In the previous studies, we found that growth factors, such as aFGF, bFGF, and PDGF, can promote wound healing [10–12]. So far, little is known about the mechanism underlying the role of FGF10 in promoting wound healing and skin function recovery. Besides, as a protein-based growth factor, FGF10 displays no sustained effect when directly applied to the wound, due to its poor stability. Poly(lactic-co-glycolic acid) (PLGA) is widely used in the drug delivery system [13]. Hence, application of PLGA for a long-term sustained release of FGF10 will be beneficial to the treatment of wound healing as well as the mechanistic studies related to FGF10.

The endoplasmic-reticulum (ER) stress response is a cellular process triggered by a variety of conditions, which disturb the protein folding in ER [14, 15]. Unreversed ER stress can impair cellular functions, often leading to cell death [16, 17]. Yuan et al. have reported that vitamin D treatment downregulates the endogenous expression of the markers of ER stress at the mRNA level, promoting wound healing in streptozotocin-induced diabetic mice [18]. We previously showed that inhibition of ER stress by other FGFs significantly improves function recovery after spinal cord injury or traumatic brain injury, suggesting a different function of FGFs in the regulation of ER stress which contributed to the recovery of multiple injuries [19, 20]. However, the role of ER stress in wound healing is still short for investigations. Given the role of regulation of ER stress mediated by aFGF, bFGF, and NGF in inhibiting apoptosis in various diseases [21–23], we hypothesized that FGF10 promotes wound healing presumably related to the regulation of ER stress.

In this study, to investigate the effect of FGF10-PLGA microsphere on wound healing, we first examined how FGF10 treatment affected the granulation formation, collagen synthesis, cell proliferation, and blood vessel density and also analyzed the role of the ER stress signaling pathway in wound healing treated with FGF10-PLGA microspheres, to further clarify the function of FGF10 and potential mechanism in the process of wound repair.

## 2. Materials and Methods

### 2.1. Reagents and Antibodies

Tunicamycin (TM) and sodium phenylbutyrate (4-PBA) were purchased from Solarbio (Beijing, China). Antibodies against p-PERK andp-IRE1*α* were from Affinity Biosciences (Zhenjiang, China); GRP78 and CD31, from Abcam (Cambridge, UK); and CHOP, PCNA, and GAPDH, from Proteintech group (Wuhan, China). The secondary antibodies (goat anti-rabbit and anti-mouse) were purchased from Beyotime (Shanghai, China). An enhanced chemiluminescence (ECL) kit was purchased from Bio-Rad (Hercules, CA, USA).

### 2.2. Preparation of FGF10-PLGA Microparticles

PLAG microspheres were prepared as described previously [24]. Slow-release microspheres loaded with FGF10-PLGA were prepared by using the traditional W/O/W compound emulsion method. Firstly, FGF10 and PLGA (50 : 50, Jinan Daigang Biomaterial Co. Ltd, China) were dissolved in deionized water and dichloromethane (DCM), respectively. Next, the polymer organic solution was mixed with FGF10 aqueous solution and emulsified by homogenization at 10000 rpm for 1 min. The prepared single emulsion was immediately emulsified by a mechanical stirrer at 400 rpn in ddH_2_O containing 0.1% *w*/*v* methylcellulose and 1% *w*/*v* PVA. Then, the above emulsion was added into 200 ml deionized water of a 0.1% *w*/*v* methylcellulose solution, followed by continued stirring for 4 hours. The PLGA microspheres were filtered, washed with water, and freeze-dried overnight.

### 2.3. Microsphere Characterization

Lyophilized PLGA microspheres were collected, sputtered with gold, and then subjected to a scanning electron microscopy analysis.

The PLGA microspheres were dissolved into PBS, and the supernatants were collected at the specific time point. After adding the fresh PBS, supernatants were quantified to assess the release of FGF10 from the PLGA microspheres by using enzyme-linked immunosorbent kits (ELISA, Westang system, China).

### 2.4. Experimental Animals and Surgical Procedures

The animal models were generated as described previously [10]. Briefly, male ICR mice at the age of 6-8 weeks, provided by Wenzhou Medical University (License No. SCXK [ZJ] 2015-0009), were anaesthetized, and hair was removed. 0.5 mm thick silicone donut shaped splints (external diameter: 16 mm, internal diameter: 8 mm) were fixed on both sides of the dorsal midline of the mice using a 6 − 0 Prolene suture. Two full-thickness cutaneous wounds were made using a 6 mm round skin biopsy punch (Acuderm Inc., Fort Lauderdale, FL, USA). The wounds in the mice were covered with Tegaderm transparent dressing (3 M Health Care, Germany) and wrapped with self-adhesive wrap to deter chewing of the splints. PBS alone, PLGA microspheres, 1.6 *μ*g of free FGF10, or FGF10-PLGA microspheres in a 20 *μ*l solution were administered to the wounds, respectively. On days 0, 3, 6, 9, 12, and 15, following the administration, Image-Pro plus for tracing the wound margin was used to measure the wound area for determining the rate of wound closure. New skin tissue was taken on the 15th day after the operation, and the number of subcutaneous vessels was observed and counted.

To assess the effect of ER stress on wound healing, the ICR mice were randomly divided into the following groups: the control group, 4-PBA group, FGF10-PLGA group, and 4-PBA + FGF10-PLGA group, or the control group (with 0.5% DMSO), TM group (with 0.5% DMSO), FGF10-PLGA group (with 0.5% DMSO), and TM + FGF10-PLGA group (with 0.5% DMSO). 4-PBA (100 mg/kg/d) or TM (0.3 mg/kg/d) (with 0.5% DMSO) was injected intraperitoneally into the mice once per day until the wound was closed completely.

### 2.5. Detection of FGF10 Release

The expression of FGF10 in the wound area was detected by enzyme-linked immunosorbent assay protocol (ELISA, Westang system, Shanghai, China). The samples were checked on the different time points.

### 2.6. In Vivo Stability Test

According to the characteristic that Cy7 (MedChemExpress, USA) can specifically bind to the lysine unit (K) in the protein amino acid sequence, FGF10 and Cy7 are formulated into a FGF10-Cy7 mixture, which is reacted on a shaker in the dark for 24 h. The dialysis technique was then used to filter out free Cy7 that was not bound to FGF10. Finally, FGF10-Cy7 were washed with water, freeze-dried overnight, and taken a part of it making to FGF10-Cy7-PLAG conjugate according to the above steps.

FGF10 and FGF10-PLAG microspheres were labeled with Cy7 and implanted into the ICR mice (FGF10-Cy7 solution/FGF10-Cy7-PLAG microspheres). At predetermined time points, the mice were anesthetized with isoflurane and imaged using a Maestro EX fluorescence image system.

### 2.7. Histological Analysis

The mice were sacrificed at the given time point after surgery, and the wounds with the surrounding tissues were collected for histological examination. The collected tissues were fixed with 4% paraformaldehyde in 0.01 M PBS at pH 7.4 overnight, embedded in paraffin, and then cut into the 5 *μ*m sections, followed by mounting on slides for the subsequent staining. Hematoxylin and eosin (H&E) staining and Masson's trichrome staining were conducted by using the staining kits from Beyotime Company (Jiangsu, China) according to the manufacturer's instructions.

### 2.8. Western Blot Analysis

For protein extraction, the tissue was homogenized in RIPA lysis buffer (1% Triton X-100, 1% deoxycholate, 0.1% SDS, 150 mM NaCl, pH 7.4) containing protease inhibitor cocktail (10 *μ*l/ml; GE Healthcare Biosciences, Pittsburgh, PA, USA). The supernatants were collected after centrifugation at 12,000 rpm for 10 min at 4°C. The protein extracts were quantified with BCA kit (Beyotime, China). Protein samples were separated by SDS PAGE electrophoresis and transferred onto polyvinylidene difluoride membranes (Bio-Rad, Hercules, CA, USA). After blocking with 5% skim milk in TBST (Tris-buffered saline with 0.1% Tween-20) for 2 h at room temperature, the membranes were incubated with primary antibodies at 4°C overnight and subsequently with horseradish peroxidase-conjugated secondary antibodies at room temperature for 1 h. Images were acquired with Chemi Doc XRS+Imaging System (Bio-Rad), and protein bands were quantified by the Quantity-One software.

### 2.9. RNA Extraction and Quantitative Real-Time PCR

Total RNA was extracted from skin tissue in 7 days after surgery, with Trizol Substitute (Solarbio, Beijing, China), following the manufacturer's protocol, and a PrimeScript RT Reagent Kit (Takara Biomedical Technology, Beijing, China) was used to synthesize cDNA. RT-PCR was performed to validate the expression pattern of selected genes using SYBR Premix EX Taq II and following the manufacturer's protocol. The primers used are listed in Table 1.

### 2.10. Immunohistochemical Staining

Wound tissue sections were fixed with 4% paraformaldehyde solution for 24 h, dehydrated in a graded series of ethanol, and embedded in paraffin. The skin tissue sections were deparaffinized, rehydrated, and then immersed in 3% H_2_O_2_ and 80% carbinol for 15 min at room temperature. The tissue sections were heated for antigen recovery in 10 mM sodium citrate buffer (pH 6.0). After washing, the samples were blocked in 5% BSA for 30 min at room temperature. The following antibodies were used in the tissue staining: rabbit polyclonal anti-PCNA antibodies, rabbit polyclonal anti-CD31 antibodies, and tagged goat anti-mouse or goat anti-rabbit secondary antibodies. After treating with a DAB chromogen kit (Beyotime, Shanghai, China), the tissue was counterstained with hematoxylin (Beyotime, Shanghai, China) for 5 min. All images were taken using a Nikon microscope.

### 2.11. Immunofluorescence Staining

The wound tissue sections were fixed with 4% paraformaldehyde solution for 24 hours, and the tissue was embedded in paraffin with ethanol gradient dehydration. The skin tissue was sliced into a thickness of 5 *μ*m, then deparaffinized and hydrated, and then immersed in 3% H_2_O_2_ and 80% methanol at room temperature for 15 minutes. The tissue sections were heated in 10 mM sodium citrate buffer (pH 6.0) to recover the antigen. After washing, the sample was blocked in 5% BSA at room temperature for 30 minutes, and the primary antibody was added overnight at 4°C. After washing with PBST, the secondary antibody was added, and finally, anti-fluorescence quencher containing DAPI for mounting was used. The following antibodies were used in tissue staining: rabbit polyclonal anti-PDI antibody (CST, USA); goat anti-rabbit IgG(H+L)-FITC (MULTI SCIENCES, China); and anti-fade mounting medium with DAPI (Beyotime, Shanghai, China).

### 2.12. Statistical Analysis

Statistically analyzed data were presented as the mean ± standard error of the mean (SEM). For the comparison of three or more groups, one-way analysis of variance (ANOVA) followed by Tukey's post hoc test was used to analyze the results. The difference was considered statistically significant when the *P* value was <0.05.

## 3. Results

### 3.1. FGF10-PLGA Microspheres Contribute to the Repair Process of Wound Healing

**Figure 1:**
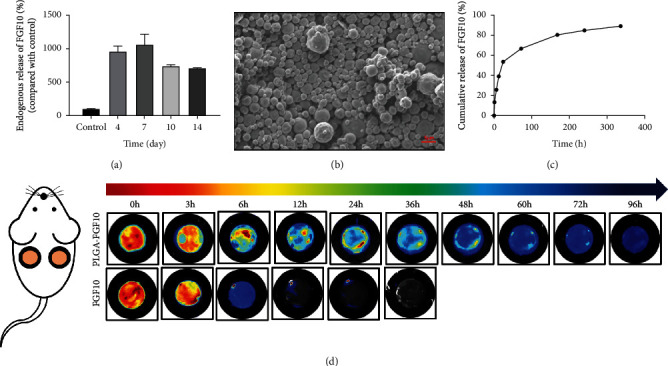
(a) The levels of FGFG10 during the wound healing at days 4, 7, 10, and 14. (b) SEM observation of PLGA microspheres. (c) Release profile of FGF10 from PLGA microspheres over a 14-day period in vitro. (d) Time-dependent near-infrared fluorescence images of FGF10-PLGA microspheres and FGF10 *in situ*.

To investigate the role of FGF10 in wound healing, we analyzed the endogenous expression of FGF10 during the process. As shown in Figure 1(a), FGF10 expression was significantly increased during wound healing and peaked at 7-day postoperation, suggesting that FGF10 may play an important role in wound healing. We next characterized FGF10-PLGA microspheres. The SEM analysis showed that FGF10-PLGA microspheres had a mean diameter of 2~4 *μ*m (Figure 1(a)). The release profiles of FGF10 from FGF10-PLGA microspheres over 14 days are shown in Figure 1(c). Then, we performed *in situ* tracing experiments on growth factors. The experiments revealed that FGF10-PLGA microspheres labeled with Cy7 (FGF10-Cy7-PLGA) lasted more than 96 h in the mouse model, while FGF10-Cy7 only lasted about 12 h (Figure 1(d)). All these results suggested that FGF10-PLGA microspheres had an ideal sustained release effect, which can have a good potential to be used in wound healing.

### 3.2. FGF10-PLGA Microspheres Accelerated Wound Closure in Mice

**Figure 2:**
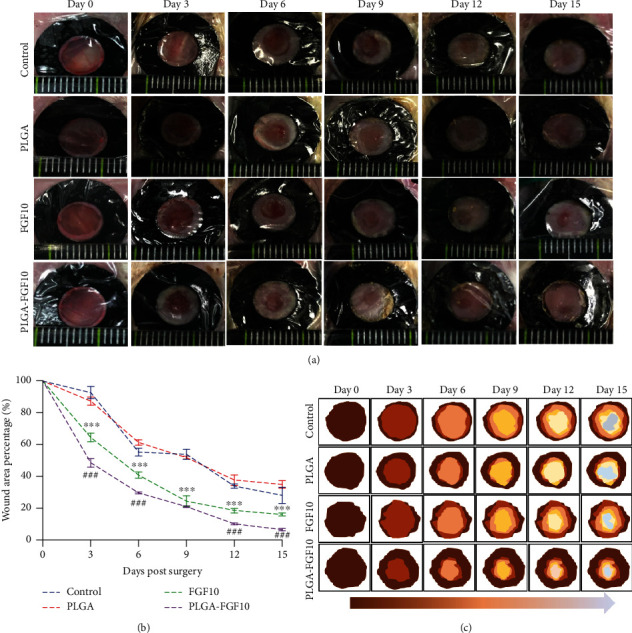
(a) Representative images of wounds of the control group, PLGA group, FGF10 group, and FGF10-PLGA microsphere group at each predetermined time point after surgery. (b) Statistical data of wound area percentages of the four groups at different time point. (c) Schematic diagram of the healing process of the three groups on days 0, 3, 6, 9, 12, and 15 after surgery. ^∗∗∗^*P* < 0.001*vs.* the control group; ^###^*P* < 0.001*vs.* the FGF10 group.

To assess the effect of FGF10-PLGA microspheres on the skin wound regeneration, different microspheres with PBS, PLGA, FGF10, and FGF10-PLGA were applied to ICR mice with the full-thick cutaneous wounds, respectively. As depicted in Figure 2(a), while there was no significant difference in wound closure between the control (PBS group) and the PLGA group, the wound closure was greatly enhanced in the groups treated with FGF10 or FGF10-PLGA microspheres compared with the negative control groups. Notably, the group of FGF10-PLGA microspheres displayed the smallest wound area among all groups, thus showing the highest wound closure rate in this experiment (Figure 2(b)). The dynamic healing process was traced in the schematic in Figure 2(c) to show a much clearer healing process. These data indicated that FGF10-PLGA microspheres exhibited a more efficient suturing effect on the wound sites, promoting wound closure throughout the healing process.

### 3.3. FGF10-PLGA Microspheres Facilitated Granulation Formation and Collagen Deposition

**Figure 3:**
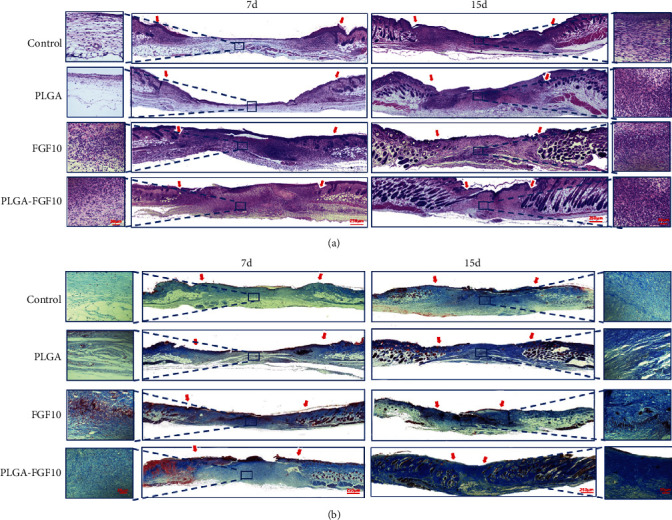
Hematoxylin and eosin (H&E) staining and Masson staining were conducted at days 7 and 15 after surgery to observe granulation formation (a) and collagen deposition (b).

Granulation tissue formation is central stage to wound repair following dermal or subcutaneous tissue injury. Granulation tissue is rich in capillaries and fibroblasts, while capillaries provide oxygen and necessary nutrients for tissue repair and fibroblasts are the main cell type involved in the tissue repair [25, 26]. H&E staining was performed on wound tissue sections treated with PBS, PLGA, FGF10, or FGF10-PLGA microspheres. As shown in Figure 3(a), the thickness of the granulation tissue was significantly different at days 7 and 15 after the treatment among all tested groups. While the control and PLGA groups displayed the thinnest granulation layers, the group of FGF10-PLGA microspheres had more continuous layers of granulation than the FGF10 groups. Meanwhile, we observed more hair follicle tissue in the group of FGF10-PLGA microspheres (day 15). We next assessed collagen deposition during the wound healing by using Masson's trichrome staining. Among all the groups, the group of FGF10-PLGA microspheres showed the highest collagen deposition, while the control and PLGA groups had the least deposition (Figure 3(b)). These results indicated that administration of FGF10-PLGA microspheres led to an enhancement in the main wound matrix of granulation and collagen formation which accelerated the process of wound healing.

### 3.4. FGF10-PLGA Microspheres Upregulated Cell Proliferative Activities and Boosted Angiogenesis

**Figure 4:**
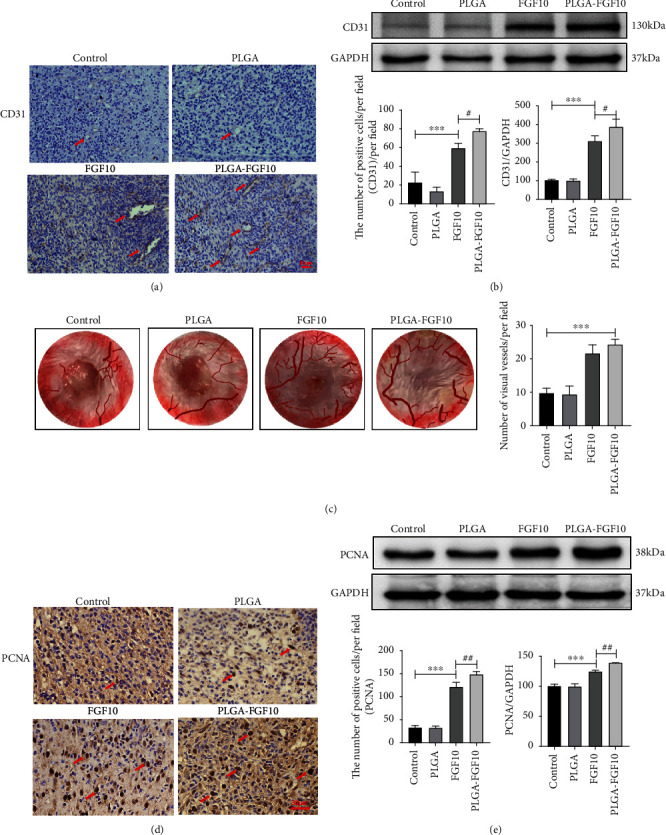
(a) Representative images of CD31 immunohistochemistry staining on day 7 after surgery. Scale bar = 30 *μ*m. (b) Protein expressions and quantification data of CD31 in each group on day 7 after surgery. ^∗∗∗^*P* < 0.001 and ^∗^*P* < 0.05. (c) Vascularization state in wound healing skin and quantification of visual vessels number on day 15 after surgery. (d) Representative images of PCNA immunohistochemistry staining on day 7 after surgery. Scale bar = 30 *μ*m. (e) Protein expressions and quantification data of PCNA in each group on day 7 after surgery. ^∗∗∗^*P* < 0.001 and ^∗^*P* < 0.05.

To examine the vascular formation during wound repair, the expression of CD31 which is a vascular endothelial cell marker was detected by western blot analysis and immunohistochemically staining, in the model mice at day 7 after the treatment. As shown in Figure 4(a), the vessel densities in wound edge were significantly higher in the group of FGF10 or FGF10-PLGA microspheres compared with the other group. Moreover, the western blot analysis revealed that the provascularization function in wound edge was significantly enhanced in the group of FGF10-PLGA microspheres, compared with the other group (Figure 4(b)). To further prove that, by directly observing the number of subcutaneous blood vessels on the 15th day, it is more intuitive to see that the blood vessel density of the PLGA-FGF10 group is significantly higher than that of the PBS group (Figure 4(c)). PCNA is characteristic by the DNA slide clamp for replicating DNA polymerase and participates in cell proliferation as an essential component of DNA replication [27]. Therefore, we can use the number of PCNA-positive spots to determine the number of cell proliferation. Next, to analyze the proliferative activities of cells during the wound healing, we detected the expression of PCNA in the mice treated with PBS, PLGA, FGF10, or FGF10-PLGA microspheres. As expected, at day 7 following the administration, more PCNA-positive cells were observed in the group of FGF10-PLGA microspheres compared with the other groups at the wound edge (Figure 4(d)). In addition, we use the western blot to detect the total expression of PCNA during wound healing at day 7. As shown in Figure 4(e), the expression of PCNA in the FGF10-PLGA group was higher than that in the FGF10 group. Additionally, the expression of PCNA in the FGF10 group was higher than that in the control group. It is known that neovascularization provides the material foundation for tissue repair, playing an important role in wound healing and tissue regeneration. Taken together, these findings demonstrated that FGF10-PLGA microspheres had effects on stimulating cell proliferation as well as angiogenesis during wound healing.

### 3.5. FGF10-PLGA Microspheres Downregulated the Expression of ER Stress-Related Proteins

**Figure 5:**
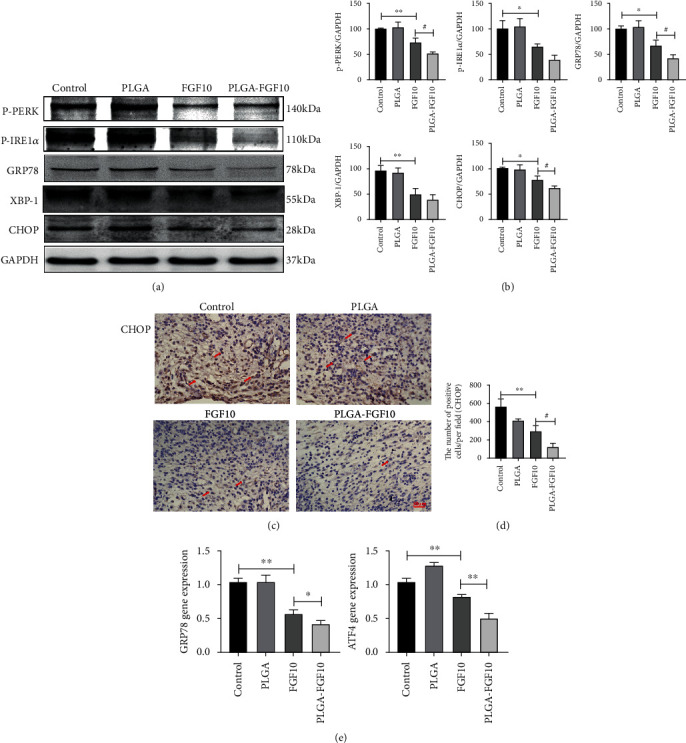
(a, b) Protein expressions and quantification data of p-PERK, p-IRE1*α*, GRP78, XBP-1, and CHOP in each group on day 7 after surgery. ^∗∗^*P* < 0.01 and ^∗^*P* < 0.05. (c, d) Immunohistochemisty staining and quantification data of CHOP in each group on day 7 after surgery. Scale bar = 30 *μ*m. (e) mRNA levels of GRP78, ATF4 in each group at day 7 after surgery. ^∗∗^*P* < 0.01 and ^∗^*P* < 0.05.

It has been shown that chronic ER stress may lead to an apoptosis, contributing to the pathophysiological processes involved in a number of prevalent disorders, including liver, kidney, neurodegenerative, and rheumatic diseases [28]. However, the potential relationship between fibroblast growth factors in wound healing and endoplasmic reticulum stress is rarely reported. To determine whether the role of FGF10-PLGA microspheres in the wound healing is related to the regulation of ER stress, we examined the expression of ER stress signaling pathway components by western blot, q-PCR, and immunohistochemical staining. As shown in Figure 5(a), the levels of the ER stress-related proteins (p-PERK, p-IRE1*α*, GRP78, XBP-1, and CHOP) were downregulated in the tissues of mice administered with FGF10 and FGF10-PLGA microspheres, and the rescue effects in the FGF10-PLGA microsphere group were better than those in the FGF10 group. Quantitatively, the expression of ER stress-related proteins is quantified in Figure 5(b); the levels of CHOP decreased to 62.54 ± 1.99 in the FGF10-PLGA microsphere group, compared with the PBS group and the FGF10 group significantly. Meanwhile, immunohistochemical staining revealed that the number of CHOP-positive cells was significantly decreased in the group of FGF10-PLGA microspheres compared with another group (Figures 5(c) and 5(d)). As is shown in Figure 5(e), the expression of GRP78 and ATF4 at the mRNA level was decreased in the FGF10 group compared with the PBS group, and their mRNA levels were much lower in the FGF10-PLGA microsphere group than in the FGF10 group. The above data suggested that the administration of FGF10-PLGA microspheres alleviated the ER stress during wound healing resulted in the accelerated recovery.

### 3.6. Tunicamycin Treatment Suppressed the Effects of FGF10-PLGA Microspheres on Wound Healing

**Figure 6:**
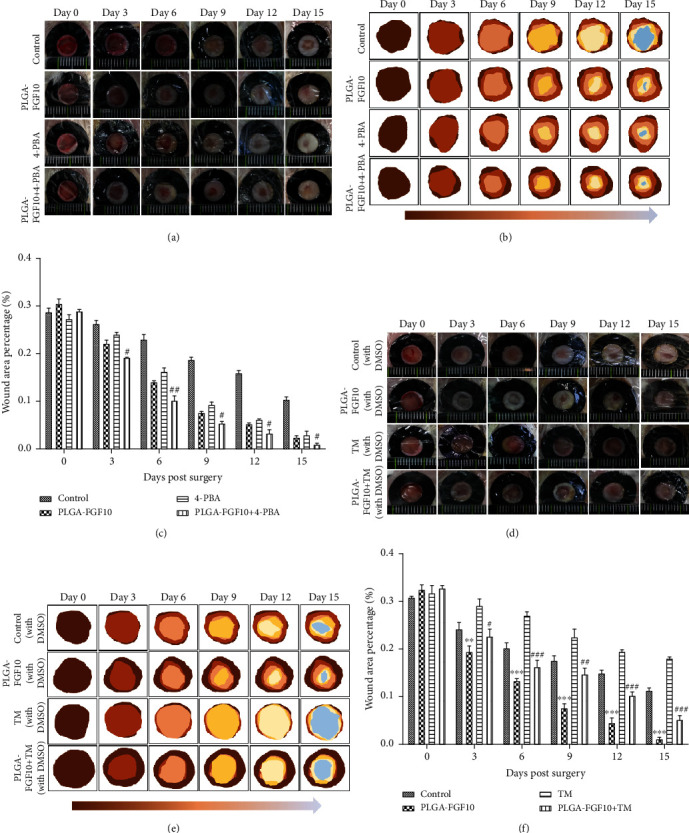
(a–c) Role of 4-PBA on wound closure in mice. ^#^*P* < 0.05 and ^##^*P* < 0.01 vs. the PLGA-FGF10 group. (d–f) The beneficial effect of FGF10-PLGA microspheres was suppressed by TM treatment. ^∗∗^*P* < 0.01 and ^∗∗∗^*P* < 0.001 vs. the TM group; ^#^*P* < 0.05, ^##^*P* < 0.01, and ^###^*P* < 0.001 vs. the TM group.

**Figure 7:**
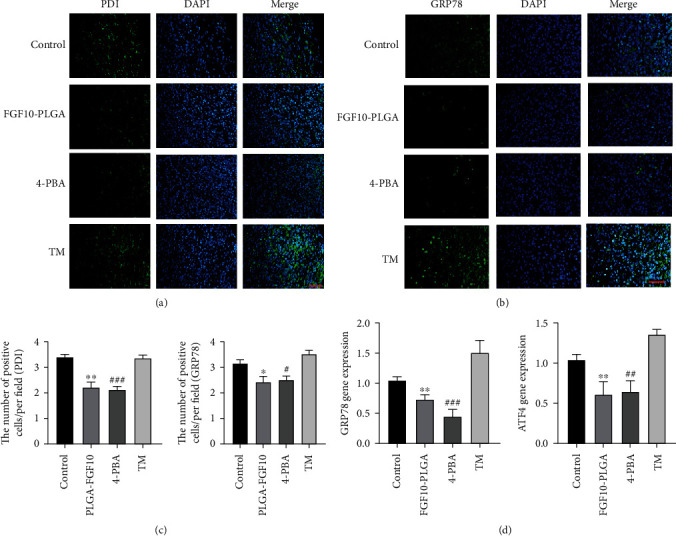
(a and b) Immunofluorescence staining of PDI and GRP78 in each group at day 7 after surgery. Scale bar = 50 *μ*m. (c) Quantification data of PDI and GRP78 in each group at day 7 after surgery. ^∗^*P* < 0.05 and ^∗∗^*P* < 0.01 vs. the TM group; ^#^*P* < 0.05 and ^###^*P* < 0.001 vs. the TM group. (d) mRNA levels of GRP78 and ATF4 in each group at day 7 after surgery; ^∗∗^*P* < 0.01 vs. the TM group; ^##^*P* < 0.01 and ^###^*P* < 0.001 vs. the TM group.

We next investigated whether FGF10-PLGA microspheres promoted wound healing through regulating the ER stress. To this end, ER stress classic activator tunicamycin (TM) and inhibitor 4-PBA were administered to the mice treated with or without FGF10-PLGA microspheres, respectively. Our data showed that 4-PBA treatment alone accelerated wound closure in the mice, while compared with FGF10-PLGA microsphere, the administration of FGF10-PLGA microspheres in combination with 4-PBA significantly promoted skin repair (Figures 6(a)–6(c)). Figures 6(d)–6(f) show that TM treatment alone inhibited wound healing compared with the FGF10-PLGA group, and wound healing speed of the FGF10-PLGA plus TM group was higher than that of the TM group. In order to further prove that FGF10 could inhibit endoplasmic reticulum stress, the protein disulfide isomerase (PDI) and glucose-regulated protein 78 (GRP78) were chosen to immunofluorescence experiments. As is shown in Figures 7(a)–7(c), the levels of ER stress (GRP78 and PDI) were lower in the FGF10-PLGA group and the 4-PBA group, compared to the TM groups. In addition, the protein (CHOP and GRP78) and gene (GRP78 and ATF4) expressions in the FGF10-PLGA group and the 4-PBA group were lower than the TM group (Figure 7(d)). Taken together, the above data suggested that FGF10-PLGA microspheres promote wound healing presumably via inhibiting ER stress.

## 4. Discussion

**Figure 8:**
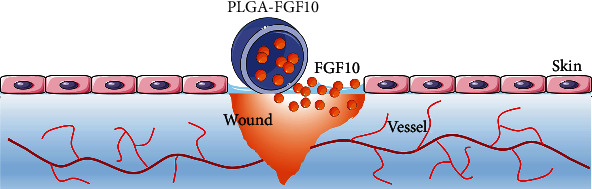
Schematic diagram of FGF10-PLGA microspheres.

Wound healing consists of four overlapping stages: hemostasis, inflammatory response period, proliferation repair phase, and wound remodeling [28]. Cell proliferation, production of extracellular matrix, and changes in cytokine concentrations are important elements in wound repair. Many types of materials are reported to be able to promote wound closure. For instance, Gou et al. reported that antibacterial hydrogels can accelerate wound healing in the infection model through inhibiting inflammation [29–31]. In recent years, with in-depth research on the healing process of wounds, growth factors have been found to be closely related to repair cells and play a key role in wound repair [32]. Upon stimulation with damage factors, cells can release multiple growth factors to activate adjacent cells or induce cell proliferation in the same germ layer involved in the reconstruction of damaged tissues and promote the repair process. In this study, we found that the level of endogenous FGF10 was increased throughout the wound healing, especially at 7 days' postoperation. In an effort to generate a drug delivery system for a long-term sustained release of FGF10, FGF10-PLGA microspheres were designed and characterized (Figure 8). Further analysis showed that the administration of FGF10-PLGA microspheres led to a long-term release of FGF10 both in *vitro* and in *vivo* (Figures 1(c) and 1(d)) as well as accelerated wound closure in the mice (Figure 2). Therefore, PLGA microspheres are of great significance for the sustained release of drugs.

It is known that FGF-10 specifically stimulates the growth of normal human epidermal keratinocytes. Jimenez and Rampy reported that FGF-10 accelerates wound healing in incisional wounds [29], but the mechanisms underlying the role of FGF-10 in wound healing remain unclear. Here, we showed that treatment with FGF10-PLGA microspheres facilitated the granulation formation and collagen deposition during the wound healing (Figures 3(a) and 3(b)). It has been shown that FGF10 stimulates HUVECs cultured on Matrigel to from capillary-like structures and induces remarkable angiogenesis response *in vivo* [30]. In a comparative study of heparin-poloxamer hydrogel modified bFGF and aFGF (HP-GFs) wound healing efficiency *in vivo*, Wu et al. explored the cell proliferation by examining the expression level of PCNA. On the 7th day, it was found that the number of PCNA-positive points in the HP-GFs group was significantly higher than that in other groups, and the wound healing in this group was also better than that in other groups [10]. Kiya and Kubo have reported that vascularization plays a vital role in the repair of skin neural networks [31], and the reformation of blood vessels has positive significance for the repair of skin wounds [33–35]. Moreover, Gou et al. have also reported that various materials used in the wound repair process can accelerate wound healing by increasing the number of blood vessels on the wound [36, 37]. Consistent with this study, FGF10 released from FGF10-PLGA microspheres upregulated cell proliferative activities by increasing the level of PCNA and boosted angiogenesis *in vivo* (Figure 4). Therefore, we proposed that FGF10-PLGA microspheres accelerate wound closure presumably through promoting granulation formation, collagen deposition, and angiogenesis.

Endoplasmic reticulum (ER) is a multifunctional organelle that coordinates protein folding, lipid biosynthesis, and calcium storage and release. A disruption of ER homeostasis leads to the misfolding of proteins, ER stress, and upregulation of a signaling pathway called ER stress response or unfolded protein response (UPR) [38]. The role of ER stress signaling during the wound healing is still unclear. ER stress is regulated by transmembrane proteins: PERK, IRE1, and ATF6 [16, 39]. Jian et al. reported that after trauma and hemorrhage, the expression of ATF6, PERK, IRE1*α*, and downstream CHOP in the liver of mice was significantly upregulated, which further indicating the existence of endoplasmic reticulum stress in the process of repair after injury [40]. And Wang et al. reported that neurotrophic factors promote the healing of corneal epithelial damage and nerve regeneration in diabetic mice by inhibiting the signal pathways related to endoplasmic reticulum stress such as XBP-1 and CHOP [41]. Recently, the ER stress proteins (PDI and GRP78) were found to be involved in the formation of pressure ulcers, and the levels of PDI and GPR78 were associated with the severity of pressure ulcers [42]. In the skin repair model, Yuan et al. reported that vitamin D can promote the healing of diabetic wound by inhibiting endoplasmic reticulum stress [18]. Yue et al. also reported that 4-PBA can reduce ischemia-reperfusion injury in the skin flap model by inhibiting endoplasmic reticulum stress of rat skin, thus increasing the survival rate of skin flap [43].

The present study revealed that ER stress was involved in wound healing treated with FGF10-PLGA microspheres (Figure 5). In this case, activation of ER stress by TM treatment reduced the therapeutic effect of FGF10-PLGA microspheres on wound healing (Figure 6). Endoplasmic reticulum stress-induced apoptosis has been implicated in the development of multiple diseases, and the ER stress and UPR are partly involved in cell apoptosis induced by translocation [44]. In our previous research, the FGF-2 could inhibit excessive ER stress by activating the PI3K/AKT and MEK-ERK1/2 signaling pathways, thereby reducing the apoptosis of renal epithelial cells induced by ischemia-reperfusion injury [45], and Li et al. have also reported that the FGF-2 could inhibit the expression of CHOP in cancer cells induced by TM [46]. Therefore, we did not exclude that the FGF10-PLGA microspheres promote wound healing through inhibiting ER stress-mediated apoptosis. In addition, further studies will be conducted to investigate the effects of FGF10 on epithelial cells as well as the relationship between ER stress and autophagy, in order to unravel the mechanisms underlying the role of FGF10 in wound healing. To further prove that the FGF10 indeed functions through the ER stress pathway to promote wound healing, CHOP conditional knockout mice should be applied to verify the function of FGF10 in the repair of skin injury.

In conclusion, we showed that the administration of FGF10-PLGA microspheres in the mice led to a long-term release of FGF10 as well as accelerated wound closure. Further analysis revealed that FGF10 released from the microspheres not only promoted granulation formation and collagen deposition but also boosted angiogenesis, enhancing the skin function recovery. Moreover, mechanistic studies suggest that FGF10-PLGA microspheres promote wound healing presumably through inhibition of ER stress related signaling, which is an important role of FGF10 in the wound healing process.

## Figures and Tables

**Table 1:** The primes of ER stress genes.

ATF4	Forward: 5′-CGGGACAGATTGGATGTTG-3′
	Forward: 5′-GGGCTCCTTATTAGTCTCTTGG-3′
GRP78	Forward: 5′-GCACTTGGAATGACCCTTC-3′
	Reverse: 5′-AAATACGCCTCAGCAGTCTC-3′

GAPDH	Forward: 5′-AGTGTTTCCTCGTCCCGTAG-3′
	Reverse: 5′-ATTTGCCGTGAGTGGAGTC-3′

## Data Availability

The data used to support the findings of this study are available from the corresponding author upon request.

## Abbreviations

FGF10: Fibroblast growth factor 10
PLGA: Poly(lactic-co-glycolic acid)
PCNA: Proliferating cell nuclear antigen
p-PERK: Phospho-protein kinase R-like endoplasmic reticulum kinase
p-IRE1*α*: Phospho-endoplasmic reticulum to nucleus signaling 1*α*
GRP78: Glucose-regulated protein 78
XBP-1: X-box binding protein-1
CHOP: C/EBP-homologous protein
ATF4: Activating transcription factor 4
PDI: Protein disulfide isomerase.
